# Electronic case report forms and electronic data capture within clinical trials and pharmacoepidemiology

**DOI:** 10.1111/bcp.13285

**Published:** 2017-04-22

**Authors:** David A. Rorie, Robert W. V. Flynn, Kerr Grieve, Alexander Doney, Isla Mackenzie, Thomas M. MacDonald, Amy Rogers

**Affiliations:** ^1^ Ninewells Hospital and Medical School University of Dundee Dundee UK

**Keywords:** electronic case report form, electronic data capture

## Abstract

**Aims:**

Researchers in clinical and pharmacoepidemiology fields have adopted information technology (IT) and electronic data capture, but these remain underused despite the benefits. This review discusses electronic case report forms and electronic data capture, specifically within pharmacoepidemiology and clinical research.

**Methods:**

The review used PubMed and the Institute of Electrical and Electronic Engineers library. Search terms used were agreed by the authors and documented. PubMed is medical and health based, whereas Institute of Electrical and Electronic Engineers is technology based. The review focuses on electronic case report forms and electronic data capture, but briefly considers other relevant topics; consent, ethics and security.

**Results:**

There were 1126 papers found using the search terms. Manual filtering and reviewing of abstracts further condensed this number to 136 relevant manuscripts. The papers were further categorized: 17 contained study data; 40 observational data; 27 anecdotal data; 47 covering methodology or design of systems; one case study; one literature review; two feasibility studies; and one cost analysis.

**Conclusion:**

Electronic case report forms, electronic data capture and IT in general are viewed with enthusiasm and are seen as a cost‐effective means of improving research efficiency, educating participants and improving trial recruitment, provided concerns about how data will be protected from misuse can be addressed. Clear operational guidelines and best practises are key for healthcare providers, and researchers adopting IT, and further work is needed on improving integration of new technologies with current systems. A robust method of evaluation for technical innovation is required.

## What is Already Known about this Subject

- Information technology (IT) has tangible benefits to assisting high quality research.
- Investment in IT is underfunded in healthcare and research.

## What this Study Adds

- A governance support framework is necessary to assist healthcare providers and researchers to maximize the benefits of IT.
- Further work is required in improving interoperability between IT systems for research and pharmacoepidemiology.
- An unambiguous legislative framework is needed to ensure high quality research can continue successfully whilst continuing to adhere to good clinical practice, data protection and ethics.
- Generic and adaptable solutions are required to meet the software needs of researchers and healthcare providers.

## Introduction

Information technology (IT) provides a fast and efficient way to collect scientific and clinical data and has become the most effective way to collaboratively share data. The benefits have underpinned the incremental introduction of electronic patient records in healthcare organizations which has been suggested as the principal reason for the increasing allocation of healthcare industry funding to IT; from 2% of total revenue, in the 1990s, to 5–7% in recent years 1. This in turn has contributed to investment in the use of IT and electronic case report forms (eCRFs) in clinical research. Whilst these systems are designed and used differently, they share a common goal of storing, and communicating in a safe and confidential way private clinical data in a structured format 2. Pharmacoepidemiology and clinical research have undoubtedly benefitted from IT; however, developments in these areas have continued to lag behind the healthcare sector, with investment limited due to various concerns. Reasons cited for not further using IT in research include: technical issues in setting up infrastructure, financing and maintaining the newest technology, and ethical fears 3. Additionally, different funding streams and personnel involved in development of electronic patient records used for healthcare purposes, and those used for data capture for research, make it difficult to integrate solutions that would satisfy both aims. The objectives of both types of system are often different, which can also lead to conflicts.

Different regulatory processes govern systems used in routine healthcare and research. However, clinical research relying on IT and electronic data capture (EDC) often depends on interfacing with healthcare IT systems, which generally comprise numerous dissimilar software systems and storage formats for storing patient data. Clinical research also often operates over large geographical areas, incorporating several different healthcare providers, further compounding challenges when interfacing with diverse local systems. Although there is a drive towards IT unification in the National Health Service primary care practises and hospital trusts in the UK are under no obligation to use collaborative IT systems or storage formats, nor are they required to make these data available for research purposes. While the need to exploit healthcare data for research to cost effectively drive healthcare improvements has never been greater, it is largely for these reasons that the task of collecting, storing and amalgamating health service data is likely to become increasingly difficult in the future.

### Objective

The objective of this review is to assess the advantages and disadvantages of eCRF and EDC technologies in pharmacoepidemiology and clinical research, and to explore where further research should be best directed. For the purpose of this paper the term eCRF will refer to a system used to capture clinical data for research and EDC will refer to the generic process of data capture.

## Methods

A literature review was conducted to identify articles pertaining to pharmacoepidemiology (drug epidemiology) and clinical research, and their use of eCRFs and EDC. Whilst the use of IT in routine healthcare is increasingly commonplace, the emphasis of this review was on the use of EDC and eCRFs in the conduct of clinical research. Common themes relating to these topics emerged covering a broad range of issues including technical and practical matters, consent, ethics, and security. PubMed and the Institute of Electrical and Electronic Engineers (IEEE) libraries were searched using to cast a wide net over the subject area; electronic case report form, eCRF, electronic data capture, and electronic data collection. Filters were applied to search terms to condense results to relevant articles (see Appendix). The search was conducted between 2014 and 2015 with a final analysis of the literature completed in August 2016. PubMed is a clinical library while IEEE is technology based.

**Figure 1:**
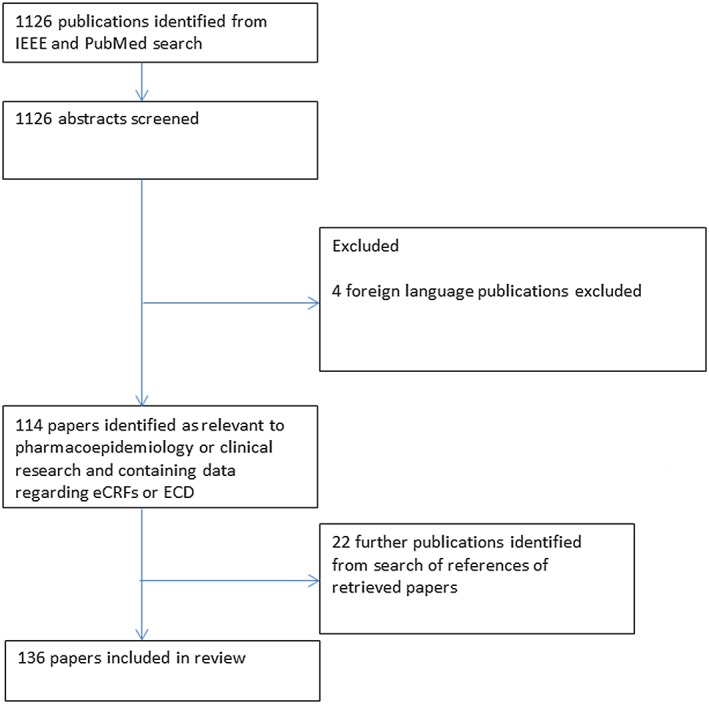
Flow diagram of review

All returned abstracts were read and articles deemed irrelevant to eCRFs and EDC, or articles that did not involve pharmacoepidemiology or clinical research, were excluded. Unlike clinical studies, IT has no universally accepted quality scoring system for academic papers. Therefore, it was decided that any published and peer reviewed article that was returned from the IEEE or PubMed search would be included. Exceptions to this were where there was an overt conflict of interest or the journal was not available in English. Figure 1 depicts a flow diagram of the review process. The authors endeavoured to adhere fully to the PRISMA checklist 4 in structuring this review; however, the nonstandard output of technical papers made this impractical. The included papers were sorted by relevance, and categorized according to whether they contained opinion or data. Papers reporting data included anecdotal data, observational data from selected data sources, observational data in population‐based studies, prospective observational data and experimental data such as clinical trials. Papers were analysed to identify reported positive and negative aspects of the IT tools being discussed.

## Results

A total of 1126 papers were returned from all search topics. After review and consideration, 136 manuscripts were deemed relevant to the review. Each topic was further separated into manuscript types. There were 17 papers documenting a study or clinical trial that used EDC where the system was the primary focus of the manuscript; 40 papers discussed observational studies comparing or evaluating EDC; 27 papers contained anecdotal evidence or opinion regarding EDC; 47 papers detailed EDC models or designs. There was one literature review, one cost benefit analysis, two feasibility studies, and one case study comparing the use of EDC in five studies (Table 1). During this review, papers were further discarded that were found to be of poor overall quality or adding little to the topic. For a list of all included publications see Table 2.

**Table 1:** Characteristics of journal papers

**Report characteristics**	***n***	**%**
**Report included one or more benefit/disadvantage of EDC**	136	
**Main objective(s) of report:**		
**Studies using EDC**	17	12.5
**Observational studies evaluating EDC use**	40	29.4
**Opinion/discussion piece**	27	19.9
**Description of model EDC system**	47	34.6
**Feasibility studies**	2	1.5
**Literature review**	1	0.7
**Cost–benefit analysis**	1	0.7
**Case study**	1	0.7

**Table 2:** Publication review list

Authors	Title	Journal	Year	Paper Type
**Aiello EJ, Taplin S, Reid R, Hobbs M, Seger D, Kamel H, *et al.***	In a randomized controlled trial, patients preferred electronic data collection of breast cancer risk‐factor information in a mammography setting 58	J Clin Epidemiol	2006	Observational
**Alexander I.**	The impact of future trends in electronic data collection on musculoskeletal research and evidence‐based orthopaedic care 71.	Arthroscopy	2003	Anecdotal
**Ariza AJ, Binns HJ, Christoffel KK, Paediatric Practice Research Group.**	Evaluating computer capabilities in a primary care practice‐based research network.	Ann Fam Med	2004	Observational
**Ashar R, Lewis S, Blazes DL, Chretien JP.**	Applying information and communications technologies to collect health data from remote settings: A systematic assessment of current technologies	J Biomed Inform	2010	Anecdotal
**Ashley L, Jones H, Thomas J, Newsham A, Downing A, Morris E, *et al.***	Integrating patient reported outcomes with clinical cancer registry data: a feasibility study of the electronic Patient‐Reported Outcomes From Cancer Survivors (ePOCS) system.	J Med Internet Res	2013	Observational
**Atreja A, Achkar JP, Jain AK, Harris CM, Lashner BA.**	Using technology to promote gastrointestinal outcomes research: a case for electronic health records.	J Gastroenterol	2008	Anecdotal
**Ayatollahi H, Mirani N, Haghani H.**	Electronic health records: what are the most important barriers? 3	Perspect Health Inf Manag	2014	Observational
**Azad T, Kalani M, Wolf T, Kearney A, Lee Y, Flannery L, *et al.***	Building an electronic health record integrated quality of life outcomes registry for spine surgery.	J Neurosur Spine	2016	Observational
**Bellamy N, Wilson C, Hendrikz J, Patel B, Dennison S.**	Electronic data capture (EDC) using cellular technology: implications for clinical trials and practice, and preliminary experience with the m‐Womac® Index in hip and knee OA patients.	Inflammopharmacology	2009	Model
**Bellary S, Krishnankutty B, Latha MS.**	Basics of case report form designing in clinical research 9.	Perspect Clin Res	2014	Model
**Bock M, Moore D, Hwang J, Shumay D, Lawson L, Hamolsky D, *et al.***	The impact of an electronic health questionnaire on symptom management and behaviour reporting for breast cancer survivors.	Breast Cancer Res Treat	2012	Observational
**Borlawsky TB, Lele O, Jensen D, Hood NE, Wewers ME.**	Enabling distributed electronic research data collection for a rural Appalachian tobacco cessation study.	J Am Med Inform Assoc	2011	Study
**Brandt CA, Cohen DB, Shifman MA, Miller PL, Nadkarni PM, Frawley SJ.**	Approaches and informatics tools to assist in the integration of similar clinical research questionnaires.	Methods Inf Med	2014	Model
**Brewster W, Gibbs T, Lacroix K, Murray A, Tydeman M, Almenoff J.**	Evolving paradigms in pharmacovigilance.	Curr Drug Saf	2006	Anecdotal
**Bruland P, Forster C, Breil B, Ständer S, Dugas M, Fritz F.**	Does single‐source create an added value? Evaluating the impact of introducing x4T into the clinical routine on workflow modifications, data quality and cost‐benefit.	Int J Med Inform	2014	Model
**Burnstead B, Furlan G.**	Unifying drug safety and clinical databases 72.	Curr Drug Saf	2013	Anecdotal
**Bushnell DM, Martin ML, Parasuraman B.**	Electronic *versus* paper questionnaires: a further comparison in persons with asthma 23.	J Asthma	2003	Observational
**Bushnell DM, Reilly MC, Galani C, Martin ML, Ricci JF, Patrick DL, *et al.***	Validation of electronic data capture of the Irritable Bowel Syndrome – Quality of Life Measure, the Work Productivity and Activity Impairment Questionnaire for Irritable Bowel Syndrome and the EuroQol.	Value Health	2003	Observational
**Carvalho JC, Bottenberg P, Declerck D, van Nieuwenhuysen JP, Vanobbergen J, Nyssen M.**	Validity of an information and communication technology system for data capture in epidemiological studies 66.	Caries Res	2011	Observational
**Cleland J, Caldow J, Ryan D.**	A qualitative study of the attitudes of patients and staff to the use of mobile phone technology for recording and gathering asthma data.	J Telemed Telecare	2007	Study
**Collins M, Ross E, Meropol NJ, Lazev AB.**	Using metadata to generate web‐based Electronic Data Capture Forms.	AMIA Annu Symp Proc	2006	Model
**Coons SJ, Gwaltney CJ, Hays RD, Lundy JJ, Sloan JA, Revicki DA, Lenderking WR, Cella D BEI ePRO TF.**	Recommendations on evidence needed to support measurement equivalence between electronic and paper‐based patient‐reported outcome (PRO) measures: ISPOR ePRO Good Research Practises Task Force report.	Value Health	2009	Model
**Courtney KL, Craven CK.**	Factors to weigh when considering electronic data collection.	Can J Nurs Res	2005	Anecdotal
**Crichton C, Davies J, Gibbons J, Harris S, Tsui A, Brenton J, *et al.***	Metadata‐driven software for clinical trials 10.	SEHC	2009	Model
**Curcin V, Soljak M, Majeed A.**	Managing and exploiting routinely collected NHS data for research 6.	Inform Prim Care	2012	Anecdotal
**Curcin V, Woodcock T, Poots AJ, Majeed A, Bell D.**	Model‐driven approach to data collection and reporting for quality improvement 17.	J Biomed Inform	2014	Model
**Dale EL, Mueller MA, Wang L, Fogerty MD, Guy JS, Nthumba PM.**	Epidemiology of operative burns at Kijabe Hospital from 2006 to 2010: pilot study of a web‐based tool for creation of the Kenya Burn Repository.	Burns	2013	Model
**Dillon DG, Pirie F, Rice S, Pomilla C, Sandhu MS, Motala AA, *et al.***	Open‐source electronic data capture system offered increased accuracy and cost‐effectiveness compared with paper methods in Africa 52.	Clin Epidemiol	2014	Observational
**Dugas M, Dugas‐Breit S, Getz K, Hearn J, Sullivan R, Stewart D, *et al.***	Integrated data management for clinical studies: automatic transformation of data models with semantic annotations for principal investigators, data managers and statisticians.	PLoS One	2014	Model
**Dunsmuir DT, Payne BA, Cloete G, Petersen CL, Görges M, Lim J, *et al.***	Development of mHealth applications for pre‐eclampsia triage.	IEEE J Biomed Heal informatics	2014	Model
**Dupont A, Wheeler J, Herndon JE, Coan A, Zafar SY, Hood L, *et al.***	Use of tablet personal computers for sensitive patient‐reported information 64.	J Support Oncol	2009	Observational
**Dy CJ, Schmicker T, Tran Q, Chadwick B, Daluiski A, Hudak PL, *et al.***	The use of a tablet computer to complete the DASH questionnaire 24.	J Hand Surg Am	2012	Observational
**Eisenstein EL, Collins R, Cracknell BS, Podesta O, Reid ED, Sandercock P, *et al.***	Sensible approaches for reducing clinical trial costs.	Clin Trials	2008	Study
**El Emam K, Jonker E, Sampson M, Krleza‐Jerić K, Neisa A.**	The use of electronic data capture tools in clinical trials: web‐survey of 259 Canadian trials.	J Med Internet Res	2009	Observational
**El Fadly A, Lucas N, Rance B, Verplancke P, Lastic P‐Y, Daniel C.**	The REUSE project: EHR as single datasource for biomedical research.	Stud Health Technol Inform	2010	Model
**El Fadly A, Rance B, Lucas N, Mead C, Chatellier G, Lastic P‐Y, *et al.***	Integrating clinical research with the Healthcare Enterprise: from the RE‐USE project to the EHR4CR platform 57.	J Biomed Inform	2011	Model
**Ene‐Iordache B, Carminati S, Antiga L, Rubis N, Ruggenenti P, Remuzzi G, *et al.***	Developing regulatory‐compliant electronic case report forms for clinical trials: experience with the demand trial 53.	J Am Med Inform Assoc	2009	Model
**Farnell DJJ, Routledge J, Hannon R, Logue JP, Cowan RA, Wylie JP, *et al.***	Efficacy of data capture for patient‐reported toxicity following radiotherapy for prostate or cervical cancer.	Eur J Cancer	2010	Observational
**Faulds MC, Bauchmuller K, Miller D, Rosser JH, Shuker K, Wrench I, *et al.***	The feasibility of using “bring your own device” (BYOD) technology for electronic data capture in multicentre medical audit and research.	Anaesthesia	2016	Feasibility Study
**Fontaine P, Mendenhall TJ, Peterson K, Speedie SM.**	The “Measuring Outcomes of Clinical Connectivity” (MOCC) trial: investigating data entry errors in the Electronic Primary Care Research Network (ePCRN).	J Am Board Fam Med	2007	Observational
**Fraccaro P, Dentone C, Fenoglio D, Giacomini M.**	Multicentre clinical trials' data management: a hybrid solution to exploit the strengths of electronic data capture and electronic health records systems.	Informatics Heal Soc Care	2013	Model
**Franklin JD, Guidry A, Brinkley JF.**	A partnership approach for Electronic Data Capture in small‐scale clinical trials 18.	J Biomed Inform	2011	Anecdotal
**Fritz F, Tilahun B, Dugas M.**	Success criteria for electronic medical record implementations in low‐resource settings: a systematic review 68.	J Am Med Inform Assoc	2015	Review
**Fu L, Ding S, Chen T.**	Clinical Data Management System.	2010 International Conference on Biomedical Engineering and Computer Science	2010	Model
**Gallagher SA, Smith AB, Matthews JE, Potter CW, Woods ME, Raynor M, *et al.***	Roadmap for the development of the University of North Carolina at Chapel Hill Genitourinary Oncology Database – UNC GOLD.	Urol Oncol	2014	Anecdotal
**Galliher JM, Stewart T V, Pathak PK, Werner JJ, Dickinson LM, Hickner JM.**	Data collection outcomes comparing paper forms with PDA forms in an office‐based patient survey.	Ann Fam Med	2008	Observational
**Gibbons C, Caudwell P, Finlayson G, King N, Blundell J.**	Validation of a new hand‐held electronic data capture method for continuous monitoring of subjective appetite sensations.	Int J Behav Nutr Phys Act	2011	Study
**Gioli‐Pereira L, Bernardez‐Pereira S, Goulart Marcondes‐Braga F, Rocha Spina JM, Muniz Miranda da Silva R, Evangelista Ferreira N, *et al.***	Genetic and Electronic medical records to predict outcomes in Heart Failure patients (GENIUS‐HF) – design and rationale.	BMC Cardiovasc Disord	2014	Study
**Goodman K, Krueger J, Crowley J.**	The automatic clinical trial: leveraging the electronic medical record in multisite cancer clinical trials 5.	Curr Oncol Rep	2012	Model
**Gupta SK.**	Paperless clinical trials: myth or reality? 2	Indian J Pharmacol	2015	Anecdotal
**Haak D, Samsel C, Gehlen J, Jonas S, Deserno TM.**	Simplifying electronic data capture in clinical trials: workflow embedded image and biosignal file integration and analysis *via* web services 11.	J Digit Imaging	2014	Model
**Haller G, Haller DM, Courvoisier DS, Lovis C.**	Handheld *vs.* laptop computers for electronic data collection in clinical research: a crossover randomized trial 67.	J Am Med Inform Assoc	2009	Observational
**Hammond WE, Bailey C, Boucher P, Spohr M, Whitaker P.**	Connecting information to improve health.	Health Aff	2010	Anecdotal
**HARDING JP, HAMM LR, EHSANULLAH RSB, HEATH AT, SORRELLS SC, HAW J, *et al.***	Use of a novel electronic data collection system in multicentre studies of irritable bowel syndrome.	Aliment Pharmacol Ther	1997	Study
**Harris PA, Taylor R, Thielke R, Payne J, Gonzalez N, Conde JG.**	Research electronic data capture (REDCap) – a metadata‐driven methodology and workflow process for providing translational research informatics support 44.	J Biomed Inform	2009	Model
**Haskew J, Kenyi V, William J, Alum R, Puri A, Mostafa Y, *et al.***	Use of Mobile Information Technology during Planning, Implementation and Evaluation of a Polio Campaign in South Sudan.	PLoS One	2015	Observational
**Hensel DJ, Fortenberry JD, Harezlak J, Craig D.**	The feasibility of cell phone based electronic diaries for STI/HIV research 65.	BMC Med Res Methodol	2012	Study
**Hetland ML.**	DANBIO – powerful research database and electronic patient record 54.	Rheumatology (Oxford)	2011	Model
**Holzner B, Giesinger JM, Pinggera J, Zugal S, Schöpf F, Oberguggenberger AS, *et al.***	The Computer‐based Health Evaluation Software (CHES): a software for electronic patient‐reported outcome monitoring 12.	BMC Med Inform Decis Mak	2012	Model
**Huffstutter J, David Craig W, Schimizzi G, Harshbarger J, Lisse J, Kasle S, *et al.***	A multicentre, randomized, open study to evaluate the impact of an electronic data capture system on the care of patients with rheumatoid arthritis.	Curr Med Res Opin	2007	Study
**Hye RJ, Inui TS, Anthony FF, Kiley M‐L, Chang RW, Rehring TF, *et al.***	A multiregional registry experience using an electronic medical record to optimize data capture for longitudinal outcomes in endovascular abdominal aortic aneurysm repair.	J Vasc Surg	2015	Study
**Installé AJ, Van den Bosch T, De Moor B, Timmerman D.**	Clinical data miner: an electronic case report form system with integrated data preprocessing and machine‐learning libraries supporting clinical diagnostic model research.	JMIR Med informatics	2014	Model
**Jamison RN, Raymond SA, Levine JG, Slawsby EA, Nedeljkovic SS, Katz NP.**	Electronic diaries for monitoring chronic pain: 1‐year validation study 69.	Pain	2001	Study
**Jamison RN, Raymond SA, Slawsby EA, McHugo GJ, Baird JC.**	Pain assessment in patients with low back pain: comparison of weekly recall and momentary electronic data 22.	J Pain	2006	Observational
**Jansen ME, Kollbaum PS, McKay FD, Rickert ME.**	Factors influencing the electronic capture of patient‐reported contact lens performance data.	Cont Lens Anterior Eye	2013	Observational
**Jensen RE, Rothrock NE, DeWitt EM, Spiegel B, Tucker CA, Crane HM, *et al.***	The role of technical advances in the adoption and integration of patient‐reported outcomes in clinical care.	Med Care	2015	Case Study
**Katzan I, Speck M, Dopler C, Urchek J, Bielawski K, Dunphy C, *et al.***	The Knowledge Program: an innovative, comprehensive electronic data capture system and warehouse.	AMIA Annu Symp Proc	2011	Model
**Kessel KA, Bohn C, Engelmann U, Oetzel D, Bougatf N, Bendl R, *et al.***	Five‐year experience with setup and implementation of an integrated database system for clinical documentation and research.	Comput Methods Programs Biomed	2014	Model
**Kho A, Zafar A, Tierney W.**	Information technology in PBRNs: the Indiana University Medical Group Research Network (IUMG ResNet) experience.	J Am Board Fam Med	2007	Anecdotal
**King C, Hall J, Banda M, Beard J, Bird J, Kazembe P, *et al.***	Electronic data capture in a rural African setting: evaluating experiences with different systems in Malawi.	Glob Health Action	2014	Model
**King JD, Buolamwini J, Cromwell EA, Panfel A, Teferi T, Zerihun M, *et al.***	A novel electronic data collection system for large‐scale surveys of neglected tropical diseases.	PLoS One	2013	Observational
**Kinnula S, Renko M, Tapiainen T, Pokka T, Uhari M.**	Post‐discharge follow‐up of hospital‐associated infections in paediatric patients with conventional questionnaires and electronic surveillance 51.	J Hosp Infect	2012	Observational
**Kohl CD, Garde S, Knaup P.**	Facilitating secondary use of medical data by using openEHR archetypes.	Stud Health Technol Inform	2010	Model
**Kuchinke W, Ohmann C, Yang Q, Salas N, Lauritsen J, Gueyffier F, *et al.***	Heterogeneity prevails: the state of clinical trial data management in Europe ‐ results of a survey of ECRIN centres.	Trials	2010	Observational
**Kush R, Alschuler L, Ruggeri R, Cassells S, Gupta N, Bain L, *et al.***	Implementing Single Source: the STARBRITE proof‐of‐concept study.	J Am Med Inform Assoc	2007	Feasibility Study
**Laird‐Maddox M, Mitchell SB, Hoffman M.**	Integrating research data capture into the electronic health record workflow: real‐world experience to advance innovation 8.	Perspect Health Inf Manag	2014	Model
**Le Jeannic A, Quelen C, Alberti C, Durand‐Zaleski I.**	Comparison of two data collection processes in clinical studies: electronic and paper case report forms 45.	BMC Med Res Methodol	2014	Observational
**Levin E, Levin A.**	Evaluation of spoken dialogue technology for real‐time health data collection.	J Med Internet Res	2006	Model
**Lin CH, Wu NY, Liou DM.**	A multi‐technique approach to bridge electronic case report form design and data standard adoption.	J Biomed Inform	2014	Model
**Long MD, Kappelman MD, Martin *CF*, Lewis JD, Mayer L, Kinneer PM, *et al.***	Development of an internet‐based cohort of patients with inflammatory bowel diseases (CCFA Partners): methodology and initial results 13.	Inflamm Bowel Dis	2012	Model
**López‐Carrero C, Arriaza E, Bolaños E, Ciudad A, Municio M, Ramos J, *et al.***	Internet in clinical research based on a pilot experience 55.	Contemp Clin Trials	2005	Model
**Lu M, Rupp LB, Moorman AC, Li J, Zhang T, Lamerato LE, *et al.***	Comparative effectiveness research of chronic hepatitis B and C cohort study (CHeCS): improving data collection and cohort identification.	Dig Dis Sci	2014	Observational
**Lu Z.**	Technical challenges in designing post‐marketing eCRFs to address clinical safety and pharmacovigilance needs 14.	Contemp Clin Trials	2010	Anecdotal
**Lu Z.**	Electronic Data‐Capturing Technology for Clinical Trials.	IEEE Engineering in Medicine and Biology Magazine	2010	Anecdotal
**Mahaffey KW, Wampole JL, Stebbins A, Berdan LG, McAfee D, Rorick TL, *et al.***	Strategic lessons from the clinical event classification process for the Assessment of Pexelizumab in Acute Myocardial Infarction (APEX‐AMI) trial.	Contemp Clin Trials	2011	Model
**Mall S, Akmatov MK, Schultze A, Ahrens W, Obi N, Pessler F, *et al.***	Web‐based questionnaires to capture acute infections in long‐term cohorts: findings of a feasibility study.	Bundesgesundheitsblatt Gesundheitsforschung Gesundheitsschutz	2014	Observational
**Maokola W, Willey BA, Shirima K, Chemba M, Armstrong Schellenberg JRM, Mshinda H, *et al.***	Enhancing the routine health information system in rural southern Tanzania: successes, challenges and lessons learned.	Trop Med Int Heal	2011	Model
**Marley JE.**	Safety and efficacy of nifedipine 20 mg tablets in hypertension using electronic data collection in general practice.	J R Soc Med	1989	Study
**Matza LS, Patrick DL, Riley AW, Alexander JJ, Rajmil L, Pleil AM, *et al.***	Paediatric patient‐reported outcome instruments for research to support medical product labelling: report of the ISPOR PRO good research practises for the assessment of children and adolescents task force.	Value Heal	2013	Anecdotal
**Meyer J, Fredrich D, Piegsa J, Habes M, van den Berg N, Hoffmann W.**	A mobile and asynchronous electronic data capture system for epidemiologic studies 15.	Comput Methods Programs Biomed	2013	Model
**Middleton RJ, Gavin AT, Reid JS, O′Reilly D.**	Accuracy of hospital discharge data for cancer registration and epidemiological research in Northern Ireland.	Cancer Causes Control	2000	Study
**Mitchel JT, Kim YJ, Choi J, Park G, Cappi S, Horn D, *et al.***	Evaluation of data entry errors and data changes to an electronic data capture clinical trial database 46.	Drug Inf J	2013	Observational
**Nahm ML, Pieper *CF*, Cunningham MM.**	Quantifying data quality for clinical trials using electronic data capture 63.	PLoS One	2008	Anecdotal
**Ndume V, Nkansah‐Gyekye Y, Lyatu I, Ko J.**	A methodology for data collection and integration of e‐Health records: A case study of Ifakara Health Institute in Tanzania.	In: 2013 Pan African International Conference on Information Science, Computing and Telecommunications (PACT)	2013	Model
**Nesbitt G, McKenna K, Mays V, Carpenter A, Miller K, Williams M, *et al.***	The Epilepsy Phenome/Genome Project (EPGP) informatics platform.	Int J Med Inform	2013	Model
**Newman ED, Lerch V, Billet J, Berger A, Kirchner HL.**	Improving the quality of care of patients with rheumatic disease using patient‐centric electronic redesign software.	Arthritis Care Res	2015	Observational
**Nichols BN, Pohl KM.**	Neuroinformatics Software Applications Supporting Electronic Data Capture, Management, and Sharing for the Neuroimaging Community.	Neuropsychol	2015	Anecdotal
**Njuguna HN, Caselton DL, Arunga GO, Emukule GO, Kinyanjui DK, Kalani RM, *et al.***	A comparison of smartphones to paper‐based questionnaires for routine influenza sentinel surveillance, Kenya, 2011–2012 21.	BMC Med Inform Decis Mak	2014	Comparative study
**Noble NE, Paul CL, Carey ML, Sanson‐Fisher RW, Blunden S V, Stewart JM, *et al.***	A cross‐sectional survey assessing the acceptability and feasibility of self‐report electronic data collection about health risks from patients attending an Aboriginal Community Controlled Health Service.	BMC Med Inform Decis Mak	2014	Observational
**Nyholm D, Kowalski J, Aquilonius S‐M.**	Wireless real‐time electronic data capture for self‐assessment of motor function and quality of life in Parkinson's disease.	Mov Disord	2004	Observational
**Ohmann C, Kuchinke W.**	Future developments of medical informatics from the viewpoint of networked clinical research.	Methods Inf Med	2009	Anecdotal
**Olsen IC, Haavardsholm EA, Moholt E, Kvien TK, Lie E.**	NOR‐DMARD data management: implementation of data capture from electronic health records.	Clin Exp Rheumatol	2014	Model
**P. O′Halloran J, S. Kemp A, P. Salmon D, N. Tariot P, S. Schneider L.**	Psychometric comparison of standard and computerized administration of the alzheimers disease assessment scale – cognitive subscale (ADASCog).	Curr Alzheimer Res	2011	Observational
**Pace WD, Staton EW.**	Electronic data collection options for practice‐based research networks.	Ann Fam Med	2005	Anecdotal
**Palmblad M, Tiplady B.**	Electronic diaries and questionnaires: designing user interfaces that are easy for all patients to use.	Qual Life Res	2004	Model
**Pavlović I, Kern T, Miklavčič D.**	Comparison of paper‐based and electronic data collection process in clinical trials: Costs simulation study.	Contemp Clin Trials	2009	Model
**Pavlovic I, Lazarevic I.**	Two models used as a basis for development of electronic data collection software to support clinical trials.	In: Twentieth IEEE International Symposium on Computer‐Based Medical Systems (CBMS'07)	2007	Model
**Pavlovic I, Miklavcic D.**	Web‐based electronic data collection system to support electrochemotherapy clinical trial 1.	IEEE Trans Inf Technol Biomed	2007	Model
**Pawellek I, Richardsen T, Oberle D, Grote V, Koletzko B.**	Use of electronic data capture in a clinical trial on infant feeding.	Eur J Clin Nutr	2012	Study
**Proctor SJ, Wilkinson J.**	A web‐based study concept designed to progress clinical research for “orphan” disease areas in haematological oncology in the elderly: the SHIELD programme.	Crit Rev Oncol Hematol	2007	Model
**Pyke‐Grimm KA, Kelly KP, Stewart JL, Meza J.**	Feasibility, acceptability, and usability of web‐based data collection in parents of children with cancer 59.	Oncol Nurs Forum	2011	Observational
**Rhodes SD, DiClemente RJ, Cecil H, Hergenrather KC, Yee LJ.**	Risk among men who have sex with men in the United States: a comparison of an Internet sample and a conventional outreach sample.	AIDS Educ Prev	2002	Study
**Salaffi F, Gasparini S, Ciapetti A, Gutierrez M, Grassi W.**	Usability of an innovative and interactive electronic system for collection of patient‐reported data in axial spondyloarthritis: comparison with the traditional paper‐administered format 62.	Rheumatology (Oxford)	2013	Observational
**SanJoaquin MA, Allain TJ, Molyneux ME, Benjamin L, Everett DB, Gadabu O, *et al.***	Surveillance Programme of IN‐patients and Epidemiology (SPINE): implementation of an electronic data collection tool within a large hospital in Malawi.	PLoS Med	2013	Model
**Sargious A, Lee SJ.**	Remote collection of questionnaires 60.	Clin Exp Rheumatol	2014	Anecdotal
**Schmier JK, Kane DW, Halpern MT.**	Practical applications of usability theory to electronic data collection for clinical trials.	Contemp Clin Trials	2005	Anecdotal
**Schreier G, Messmer J, Rauchegger G, Modre‐Osprian R, Ladenstein R.**	A Web‐based platform for interdisciplinary biomedical research 42.	Front Biosci (Landmark Ed)	2009	Model
**Schrimpf D, Haag M, Pilz LR.**	Possible combinations of electronic data capture and randomization systems. Principles and the realization with RANDI2 and OpenClinica.	Methods Inf Med	2014	Anecdotal
**Shah J, Rajgor D, Pradhan S, McCready M, Zaveri A, Pietrobon R.**	Electronic data capture for registries and clinical trials in orthopaedic surgery: open source *versus* commercial systems.	Clin Orthop Relat Res	2010	Anecdotal
**Taylor MJ, Stables R, Matata B, Lisboa PJG, Laws A, Almond P.**	Website design: technical, social and medical issues for self‐reporting by elderly patients 16.	Health Informatics J	2014	Observational
**Thriemer K, Ley B, Ame SM, Puri MK, Hashim R, Chang NY, *et al.***	Replacing paper data collection forms with electronic data entry in the field: findings from a study of community‐acquired bloodstream infections in Pemba, Zanzibar 47.	BMC Res Notes	2012	Observational
**Thwin SS, Clough‐Gorr KM, McCarty MC, Lash TL, Alford SH, Buist DSM, *et al.***	Automated inter‐rater reliability assessment and electronic data collection in a multi‐center breast cancer study 50.	BMC Med Res Methodo	2007	Study
**Trachtenberg FL, Martin M, Green S, Oliveros O, Carson S, Gerstenberger E, *et al.***	Use of electronic data collection to assess pain in thalassaemia: a feasibility study.	Int J Palliat Nurs	2012	Observational
**Vahabzadeh M, Mezghanni M, Gupman AE, Schmittner J, Preston KL.**	An adaptable assessment generation system for clinical trials complementing human research information system 43.	18th IEEE Symposium on Computer‐Based Medical Systems (CBMS'05)	2005	Model
**Walther B, Hossin S, Townend J, Abernethy N, Parker D, Jeffries D.**	Comparison of electronic data capture (EDC) with the standard data capture method for clinical trial data 48.	PLoS One	2011	Observational
**Wang SJ, Middleton B, Prosser LA, Bardon CG, Spurr CD, Carchidi PJ, *et al.***	A cost–benefit analysis of electronic medical records in primary care 56.	Am J Med	2003	Cost benefit analysis?
**Weiler K, Christ AM, Woodworth GG, Weiler RL, Weiler JM, Meltzer E, *et al.***	Quality of patient‐reported outcome data captured using paper and interactive voice response diaries in an allergic rhinitis study: is electronic data capture really better?	Ann Allergy, Asthma Immunol	2004	Observational
**Welker JA, Cooper‐Dehoff R, Handberg E, Heissenberg C, Johnson K, Hyde AW, *et al.***	Implementation of electronic data capture systems: barriers and solutions.	Contemp Clin Trials	2007	Anecdotal
**Whalen CJ, Donnell D, Tartakovsky M.**	Supporting research sites in resource‐limited settings: challenges in implementing information technology infrastructure.	J Acquir Immune Defic Syndr	2014	Anecdotal
**Wildeman MA, Zandbergen J, Vincent A, Herdini C, Middeldorp JM, Fles R, *et al.***	Can an online clinical data management service help in improving data collection and data quality in a developing country setting? 49	Trials	2011	Observational
**Wintner LM, Giesinger JM, Zabernigg A, Rumpold G, Sztankay M, Oberguggenberger AS, *et al.***	Evaluation of electronic patient‐reported outcome assessment with cancer patients in the hospital and at home.	BMC Med Inform Decis Mak	2015	Observational
**Xu W, Guan Z, Sun J, Wang Z, Geng Y.**	Development of an open metadata schema for prospective clinical research (openPCR) in China.	Methods Inf Med	2014	Model
**Yamamoto K, Yamanaka K, Hatano E, Sumi E, Ishii T, Taura K, *et al.***	An eClinical trial system for cancer that integrates with clinical pathways and electronic medical records 7.	Clin Trials	2012	Model
**Yuksel UC, Anabtawi AGM, Cam A, Poddar K, Agarwal S, Goel S, *et al.***	Predictive value of renal resistive index in percutaneous renal interventions for atherosclerotic renal artery stenosis.	J Invasive Cardiol	2012	Study
**Zbrozek A, Hebert J, Gogates G, Thorell R, Dell C, Molsen E, *et al.***	Validation of electronic systems to collect patient‐reported outcome (PRO) data—recommendations for clinical trial teams: report of the ISPOR ePRO systems validation good research practises task force.	Value Health	2013	Anecdotal

Research has been conducted into ways to maximize data accuracy and efficiency using IT. Trials have taken data from patient's electronic medical records (EMRs) and transferred these directly into eCRFs. The cost savings, quality improvements, and reduction of data entry errors, were significant 5, 6, 7. Whilst not all required data ARE available from the patient EMR, studies have found varying results with as much as 69% of data required being found and used to prepopulate trial eCRFs 8. Discussions around the design and theoretical modelling of EMRs, eCRFs and ECD were prevalent within the included papers 9, 10, 11, 12, 13, 14, 15, 16, 17. The electronic systems reported vary in quality, with some being used in mock environments and others being purely theoretical. Commercial software packages are available, but are generally not cost effective and in some circumstances it is unclear who owns the data entered into them 18, 19, 20. Observational studies have compared paper based systems against EDC or canvassed opinion on the use of EDC systems 21, 22, 23, 24. These papers were overwhelmingly in favour of EDC as long as security could be maintained.

Obtaining patient consent is an ethical necessity, and up until recently, has almost always required a physical signature. Varnhagen *et al.* 25 considered obtaining informed consent online and questioned whether it is ethical to obtain consent electronically. Recently, electronic consent has been accepted by the National Health Service as a viable alternative to a written signature 26. This review found one trial where consent had successfully been captured online 27. Collecting participant consent electronically is a novel, and largely unexplored, method that invites further innovation. There are ethical implications of conducting research entirely online. IT is advancing faster than ethical review panels can address and there is a need for greater ethical consideration of conducting research online and how we share data between IT systems and within organizations 28, 29, 30, 31, 32, 33, 34, 35. Government attempts to legislate – the Health Insurance and Portability Act 36 in the USA, and the Data Protection Act 37 in the UK, have had little impact on alleviating public scepticism 38. Patient privacy is critical and despite the well‐intentioned zeal for the mass adoption of IT within healthcare, serious security concerns remain 39, 40. However, patients are open to technology being used to store their medical data if trust and privacy concerns can be addressed 41.

Clinical research and pharmacoepidemiology often involve interdisciplinary research. This not only means that various researchers deal with different data sources and formats but also that they have different workflows and organizational structures. As a consequence, there are no off‐the‐shelf solutions to facilitate this. This often results in individual solutions being developed that, over time, evolve and are ultimately difficult to maintain 42. Unfortunately, it is often much easier to change time points, interventions, and assessment tools on paper than it is to suddenly change the programming of a computerized system. The reality demands future IT systems be flexible and adaptable with more automation 43.

### Advantages and disadvantages

There are distinct advantages to EDC in research and pharmacoepidemiology. However, there are pragmatic concerns that need to be addressed. The role of clinical research and pharmacoepidemiology is to improve healthcare by generating and providing access to high quality data. Due to the limitations of paper based records this is not possible with the status quo 44. The objectives of ECD are to reduce medical errors, improve communication between healthcare providers, collect information for educational and research purposes and to gather complete and accurate data whilst avoiding duplication.

EDC's distinct advantage over paper‐based systems of research is that it is able to detect protocol violations and data outside the normal range at the time of entry and not days, weeks or months after. EDC systems have been shown to improve the quality of clinical trials, halt the development of ineffective or unsafe drugs earlier, reduce unnecessary work, reduce cost, and accelerate time to market of new drugs 45, 46, 47, 48, 49, 50, 51. There are also benefits in relation to data quality, performance, productivity and costs in clinical trial management 52, 53, 54, 55, 56. Observational data suggest that it is now considered a preferred method of data capture in clinical research 57, 58, 59. It is well accepted by users and has been shown to contribute to patient empowerment, allowing them to be more engaged in research and to take direct control of their own data 60, 61, 62. By contrast, paper‐based questionnaires can suffer from incomplete forms, questions being answered twice or skipped questions. Paper forms are considered time consuming, require dual checking, and data cleansing 63, whereas EDC can alert people to missing answers before any attempt to proceed, and can be easily incorporated into electronic health records. Remote data collection offers the additional advantage of convenience to patients, particularly those who are incapacitated or live far from the nearest clinic 47, and may provide a safer environment for questionnaires than paper‐based methods eliciting the answers to potentially sensitive questions 64, 65.

Despite the advantages, EDC has not been universally accepted. Perceived disadvantages and concerns regarding EDC include: a lack of available technical support, a lack of investigator motivation, complexity of installation, maintenance of software, high initial investment cost, and complexity of use 66, 67. Reliable data handling methods, effective project management, and expert technical architecture and infrastructure are all key factors for successful implementation, and should not be underestimated 68. There are concerns over patient privacy and the need for computer literacy, which may affect generalisability of any research findings 60. Study retention is considered to be higher where there is direct patient interaction because of the explicit alignment of patient incentives; the patient learns about the study directly, understands what is required, self‐consents to participate, and then self‐reports study information 69. Jamison *et al.* 70 found better rates of compliance with electronic patient reported outcomes (PROs) than paper based PROs. Despite data suggesting benefits of EDC use, Alexander 71 reports that physicians lack motivation and will only use structured electronic records if the system reduces overhead while at the same time minimizing their work load. In the UK, it has been suggested that development of these technologies suffers from the lack of a clear national direction towards unifying clinical and medical data, with no common format for all data systems. Not only would EDC benefit clinical research, but pharmacovigilance and drug safety regulation could also be improved 72.

## Discussion

IT and how it is used in pharmacoepidemiology and clinical research is a relatively new field with no substantial guidelines in place and few recommendations. There is a consensus that EDC has clear benefits for use in research but there are fears over security and data protection which must be addressed. IT offers an opportunity to improve pharmacoepidemiology and clinical research and to facilitate the continual improvement of healthcare. If the use of IT in research is to succeed fully, change is required: specifically, investment in infrastructure and the provision of support for integration of interoperable systems. Further efforts will be essential to alleviate healthcare providers and users legitimate concerns regarding IT. Policy makers will need to find ways to supply adequate financial resources to IT to counter a historical lack of investment within the public sector.

Healthcare providers and researchers require a governance‐led support network of technology experts to assist in integrating ever more complex systems and providing guidance on compliance and security. IT security is a challenging and fast moving field and requires careful consideration. There is a need for clearer and more consistent policies and more trained data managers, software architects and semantic web specialists working in medical research groups. These architectures will need ongoing support from a robust legal system protecting patient privacy.

Furthermore, the exchange of information between systems is essential. Data format differences need to be resolved, and a solution found for interoperability between healthcare systems. Motivating software vendors, healthcare providers and researchers to agree on a common path will be difficult but worthwhile endeavour. Future technical development needs to focus on creating adaptable and generic software that can be tailored to specific trial needs without major re‐development.

### Limitations

This work has several limitations. Firstly, a publication bias is very likely as less successful IT projects are unlikely to be reported in published literature. This review only searched the IEEE library and the PubMed database and we did not include papers in non‐English languages. In addition, researchers from low‐income countries are known to have lower publication rates. The relative novelty of the field means that evaluation studies, in particular, are missing and rapid developments in the field may not yet have been published at the time of conducting the literature search. There are currently no widely accepted methods to evaluate technical publications in the same way as has been developed for reports of clinical trials, for example. Therefore, subjective interpretation had to be used to decide if a journal was of sufficient quality to be referenced. The authors took steps to avoid selection and objectivity bias by including all peer reviewed and published articles. The only exceptions authors made were where there was an overt conflict of interest, or the journal was not available in English. This review aimed to capture the full range of reported advantages and benefits of IT use. It did not measure the relative frequency or impact of individual factors of the utility of EDC and eCRFs. Despite the limitations detailed above, the authors believe this review to be an unbiased appraisal of publications on EDC and eCRFs in pharmacoepidemiology and clinical research.

## Conclusion

It is apparent from the results of this literature review that the following areas would benefit from further development:

- Clearer legislation and operational frameworks governing electronic health records.
- Guidelines and best practises for researchers to follow in the use of IT and EDC.
- Standard methods of reporting and evaluating technical innovation to facilitate comparison.

Regardless of the challenges, it is the imperative that healthcare organizations ensure that patients receive safe medications. Effective clinical research and pharmacoepidemiology are essential to this process.

## Competing Interests

There are no competing interests to declare.

*The authors acknowledge the support of the Medicines Monitoring Unit, University of Dundee.*

## Contributors

D.R. conceived the idea with input from T.M.. The initial draft of the manuscript was created by D.R. T.M., R.F., A.D., K.G., I.M. and A.R. analysed and reviewed the manuscript. All listed authors fulfil the requirements for authorship and agree to submission of the manuscript in its current form.

## Appendix

**Table A1:** Electronic data capture – IEEE

**Search term used**	**Results**	**Search criteria and filters**	**Date**
**electronic case report form**	221	NA	27/07/2016
**(“Abstract”: electronic data capture)**	18	NA	27/07/2016
**ecrf**	14	NA	27/07/2016
**(“Abstract”: electronic data collection)**	15	NA	27/07/2016

**Table A2:** Electronic data capture – PubMed

**Search term used**	**Results**	**Search criteria and filters**	**Date**
**(electronic data capture[Title/Abstract]**)	170	Abstract available, Humans	27/07/2016
**electronic case report form**	546	Abstract available, Humans	27/07/2016
**ecrf**	20	Abstract available, Humans	27/07/2016
**(electronic data collection[Title/Abstract])**	122	Abstract available, Humans	27/07/2016
